# MicroRNA-155 induction via TNF-α and IFN-γ suppresses expression of programmed death ligand-1 (PD-L1) in human primary cells

**DOI:** 10.1074/jbc.M117.809053

**Published:** 2017-10-24

**Authors:** Daniel Yee, Kunal M. Shah, Mark C. Coles, Tyson V. Sharp, Dimitris Lagos

**Affiliations:** From the ‡ Centre for Immunology and Infection, Department of Biology and Hull York Medical School, University of York, York YO10 5DD and; the § Centre for Molecular Oncology, Barts Cancer Institute, Queen Mary University of London, London EC1M 6BQ, United Kingdom

**Keywords:** endothelial cell, fibroblast, inflammation, interferon, microRNA (miRNA), PD-L1, immune checkpoint inhibitors, lymphatic endothelial cells, miR-155

## Abstract

Programmed death ligand-1 (PD-L1) is a critical regulator of T cell function contributing to peripheral immune tolerance. Although it has been shown that posttranscriptional regulatory mechanisms control PD-L1 expression in cancer, it remains unknown whether such regulatory loops operate also in non-transformed cells. Here we studied PD-L1 expression in human dermal lymphatic endothelial cells (HDLECs), which play key roles in immunity and cancer. Treatment of HDLECs with the pro-inflammatory cytokines IFN-γ and TNF-α synergistically up-regulated PD-L1 expression. IFN-γ and TNF-α also affected expression of several microRNAs (miRNAs) that have the potential to suppress PD-L1 expression. The most highly up-regulated miRNA following IFN-γ and TNF-α treatment in HDLECs was miR-155, which has a central role in the immune system and cancer. Induction of miR-155 was driven by TNF-α, the effect of which was significantly enhanced by IFN-γ. The PD-L1 3′-UTR contains two functional miR-155-binding sites. Endogenous miR-155 controlled the kinetics and maximal levels of PD-L1 induction upon IFN-γ and TNF-α treatments. We obtained similar findings in dermal fibroblasts, demonstrating that the IFN-γ/TNF-α/miR-155/PD-L1 pathway is not restricted to HDLECs. These results reveal miR-155 as a critical component of an inflammation-induced regulatory loop controlling PD-L1 expression in primary cells.

## Introduction

Lymphatic endothelial cells (LECs)^4^ line the vessels of a large network that regulate the traffic of immune cells and antigen to lymph nodes, which orchestrate the adaptive immune response (1). The constant interaction between lymphatic vessels and the immune system enables the lymphatic system to serve as an important conduit in inflammation, infection, wound healing, and cancer. The inflammatory response can be regulated by the expression of inhibitory immune checkpoint proteins such as programmed cell death protein-1 (PD-1) and its ligand PD-L1, which act to suppress T cell activation and induce peripheral tolerance (2). Recently, immune restoration through PD-L1 blockade has shown remarkable efficacy and improvement in the treatment of solid cancers (3, 4). PD-L1 is widely expressed on immune cells including T cells, B cells, macrophages, and dendritic cells. Interestingly, expression of PD-L1 has also been detected in murine LECs and can facilitate deletional or dysfunctional tolerance of CD8^+^ T cells (5–8). However, little is known about the regulation of PD-L1 under an inflammatory environment in endothelial cells. Interferon-γ (IFN-γ) and tumor necrosis factor-α (TNF-α) are key inducers of PD-L1 expression (9–11). Of note, the 3′-untranslated region (UTR) is a crucial determinant of PD-L1 expression. Whole genome sequencing in adult patients with T cell leukemia/lymphoma or B cell lymphoma revealed structural variations that disrupted the 3′-UTR of the PD-L1 gene (12). These variations led to truncation of the 3′-UTR resulting in elevated mRNA levels of PD-L1. A mouse tumor model with CRISPR-Cas9 deletion of the 3′-UTR confirmed an increase in PD-L1 expression compared with wild-type, which could be synergistically up-regulated with IFN-γ stimulation (12). This is thought to be at least partly due to the activity of miRNAs, which are small (20–24 nucleotides), highly conserved, single-stranded non-coding RNAs that regulate gene expression at the posttranscriptional level. miRNAs regulate a wide range of developmental and cellular processes in eukaryotic organisms by directly binding to the 3′-UTRs of target mRNAs to repress protein expression (13). miRNAs are dysregulated in disease and can be used in the clinic as biomarkers through detection in biological fluids (14). Studies have demonstrated miRNAs in the regulation of inflammation including miR-146a/b, miR-155, and miR-132 (15–18). In parallel, several miRNAs, including miR-200, miR-34a, and miR-138, have been found to be down-regulated in cancer cells to allow PD-L1 expression (19–21). However, it remains unknown whether miRNAs contribute toward PD-L1 regulation in human primary cells responding to inflammatory stimuli.

Here we show that PD-L1 is expressed in primary human dermal LECs (HDLECs) and IFN-γ and TNF-α act synergistically to induce PD-L1 expression in these cells. Using this cellular model of inducible PD-L1 expression we distinguish a number of potential PD-L1-targeting miRNAs. We identify differentially regulated miRNAs upon IFN-γ and TNF-α stimulation of HDLECs and show that miR-155 is the most highly up-regulated miRNA. Furthermore, we show that there are two functional miR-155-binding sites on the 3′-UTR of PD-L1. Mutation of both of these binding sites results in de-repression of a reporter under control of the PD-L1 3′-UTR. Consistent with these findings, miR-155 overexpression or inhibition results in suppression or enhancement of PD-L1 protein expression, respectively. Similar effects can also be observed in primary human dermal fibroblasts (HDFs), indicating that the IFN-γ/TNF-α/miR-155/PD-L1 regulatory loop is not restricted to HDLECs. These results suggest that during physiological immune activation of HDLECs, IFN-γ and TNF-α synergize to induce PD-L1 expression and concurrently activate miRNA networks that suppress PD-L1 expression, presumably to avoid prolonged immune suppression. Overall, our study reveals how the HDLEC small RNA landscape responds to inflammation and provides new insight into posttranscriptional regulation of PD-L1 in human primary cells.

## Results

### PD-L1 is expressed in LECs and can be synergistically induced by IFN-γ and TNF-α

**Figure 1.**
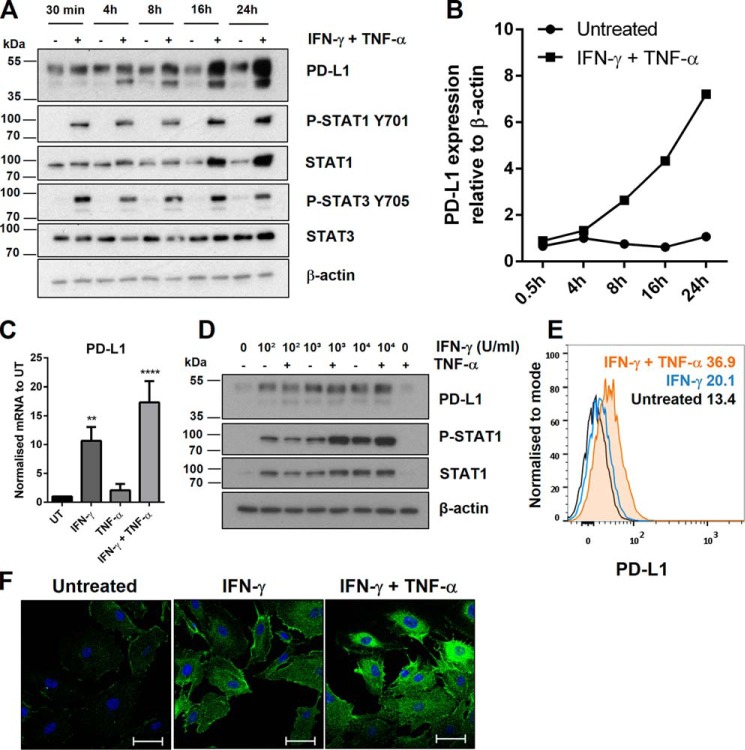
**PD-L1 is expressed in LECs and can be synergistically induced by IFN-γ and TNF-α.** *A,* Western blot analysis following a time course of IFN-γ and TNF-α-stimulation in HDLECs. *B,* Western blot quantification of PD-L1 expression from *A* in untreated and IFN-γ- and TNF-α-treated samples, relative to β-actin. *C,* PD-L1 mRNA levels measured by qRT-PCR after stimulation (24 h) and normalized to untreated (*UT*). One-way analysis of variance was calculated with Tukey's multiple comparisons test. **, *p* < 0.01 and ****, *p* < 0.0001. *D,* protein expression following titration of IFN-γ stimulation (24 h) with or without TNF-α. *E,* flow cytometric analysis showing PD-L1 surface expression (median fluorescence intensity) after stimulation (24 h) with IFN-γ alone (*blue*), or IFN-γ with TNF-α (*orange*). *F,* immunofluorescence microscopy showing PD-L1 (Alexa Fluor 488) in HDLECs after stimulation (24 h) with IFN-γ, or in combination with TNF-α. Cells were permeabilized prior to staining. DAPI is shown to mark the nucleus. *Scale bar* = 50 μm.

Human macrovascular endothelial cells become activated by pro-inflammatory cytokines IFN-γ and TNF-α and display increased PD-L1 expression (5, 22). However, the expression profile of PD-L1 in HDLECs has not been validated. PD-L1 expression was measured at basal levels and at different time points following IFN-γ and TNF-α stimulation. PD-L1 expression was induced after 4 h of stimulation and this up-regulation was increased further by 24 h (Fig. 1, *A* and *B*). IFN-γ is capable of activating both signal transducer and activator of transcription 1 and 3 (STAT1/3). Both STAT1 and STAT3 have been shown to contribute toward increased PD-L1 expression (23, 24). In HDLECs, phosphorylation of STAT1 at the activating tyrosine residue (Tyr-701) correlated with the increase of PD-L1, whereas the kinetics of STAT3 activation were transient but remained induced. Two protein bands were observed for PD-L1 (around 40–50 kDa). Transfection of small interfering RNA (siRNA) targeting PD-L1 abolished detection of both bands (supplemental Fig. S1*A*). PD-L1 is predicted to have up to four *N*-linked glycosylation sites. De-glycosylation treatment led to total disappearance of both bands and a new band appearing at 33 kDa, which is the expected molecular mass of unmodified PD-L1 (supplemental Fig. S1*B*). Consistently with induction of the JAK/STAT pathway we showed that PD-L1 mRNA levels were up-regulated in activated HDLECs. IFN-γ treatment resulted in a 10-fold induction of PD-L1 mRNA levels at 24 h post-treatment (Fig. 1*C*). The effect was significantly elevated by concurrent addition of TNF-α, although addition of TNF-α alone did not significantly affect PD-L1 mRNA levels, demonstrating that the effect of stimulating with both cytokines was synergistic. Similarly, increasing the concentration of IFN-γ stimulation led to up-regulation of PD-L1 mRNA, which was further augmented in combination with TNF-α (supplemental Fig. S1*C*). Expression of interleukin-1β (IL-1β) mRNA, a downstream target of TNF-α signaling through the NF-κB pathway, was up-regulated in a similar synergistic manner between IFN-γ and TNF-α (supplemental Fig. S1*D*). PD-L1 mRNA was measured at 8 h where it was strongly induced and remained at the same level at 24 h, consistent with the cumulative increase in PD-L1 protein levels (supplemental Fig. S1*E*). Although TNF-α stimulation alone induced a minor increase in PD-L1 mRNA levels, protein expression of PD-L1 did not change compared with untreated levels (Fig. 1*D*). However, addition of TNF-α to IFN-γ-treated cells enhanced surface PD-L1 expression (Fig. 1*E*), whereas having minimal effects on total PD-L1 protein levels in comparison to treatment with IFN-γ alone (Fig. 1*D*). This indicated that, in IFN-γ-treated HDLECs, TNF-α can affect PD-L1 localization and mRNA levels (Fig. 1*C*). The effects of IFN-γ and TNF-α on PD-L1 expression were also determined by immunofluorescence. PD-L1 was localized at the cell membrane and throughout the cytoplasm and its levels increased following stimulation with the cytokines (Fig. 1*F*). Taken together, these data indicated that, as in the case of macrovascular endothelial cells, PD-L1 is inducible at the transcriptional level in HDLECs responding to inflammatory stimuli.

### Small RNA sequencing of IFN-γ and TNF-α-stimulated LECs reveal inflammation-responsive miRNAs

**Figure 2.**
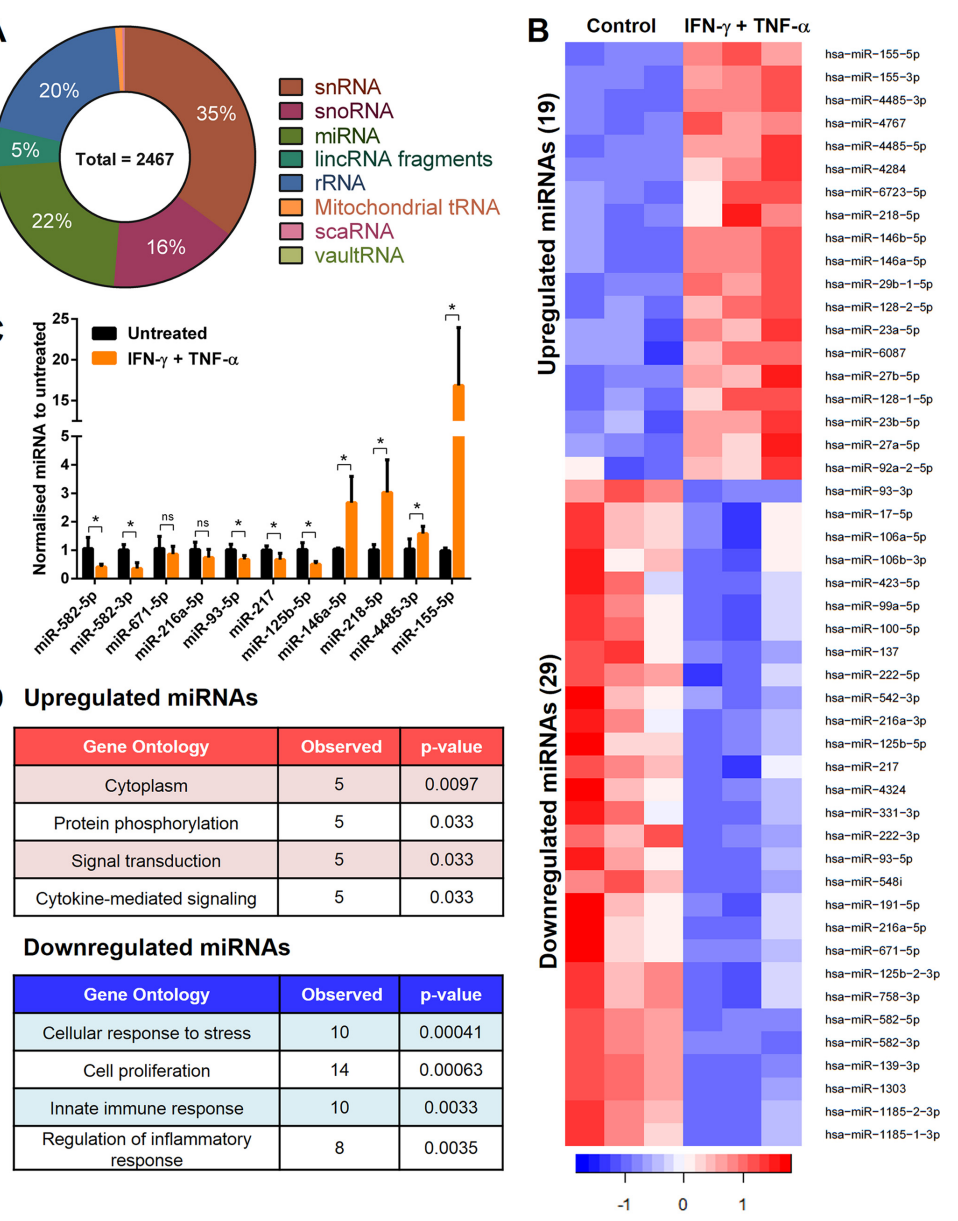
**Small RNA sequencing of IFN-γ and TNF-α-stimulated LECs reveal inflammation-responsive miRNAs.** *A,* percentage distribution of sequencing results from HDLECs, showing the total number of hits after a threshold to filter lowly expressed genes was applied (>50 RPKM). *B,* heat map showing fold-change in expression of 48 miRNAs after IFN-γ and TNF-α stimulation (24 h) in HDLECs (adjusted *p* < 0.1). Row Z-score represents mean ± S.D., *n* = 3 independent samples performed in triplicate. *C*, validation of selected IFN-γ- and TNF-α-regulated miRNAs targets by qRT-PCR. Statistical analysis by unpaired Student's *t* test, *, *p* < 0.05, *n* = 3 independent samples. *D,* gene ontology analysis of 48 IFN-γ and TNF-α-regulated miRNAs.

Having shown that PD-L1 is inducible in HDLECs responding to inflammatory stimuli, we reasoned that this was an appropriate cellular model for identifying posttranscriptional PD-L1 regulators during inflammatory responses of primary human cells. To this aim and as the small RNA transcriptome of IFN-γ- and TNF-α-treated HDLECs had not been determined, we analyzed small non-coding RNAs in HDLECs stimulated with or without IFN-γ and TNF-α for 24 h. Collected RNA were enriched for small RNAs and analyzed on an Illumina MiSeq. Sequencing detected small nuclear RNAs (snRNAs), small nucleolar RNAs (snoRNAs), and miRNAs (Fig. 2*A*). More specifically, 48 miRNAs were identified to be differentially regulated by IFN-γ and TNF-α (adjusted *p* < 0.1) (Fig. 2*B*). Levels of up-regulated and down-regulated miRNAs were further assessed by qRT-PCR (Fig. 2*C*). We found that IFN-γ and TNF-α resulted in up-regulation of miR-155–5p, miR-4485–3p, miR-218–5p, and miR-146a–5p and down-regulation of miR-582–5p, miR-582–3p, miR-93–5p, miR-217 and miR-125b–5p (supplemental Tables S1 and S2). Gene ontology analysis using the miRNA enrichment analysis and annotation tool (25) indicated that predicted targets of these differentially regulated miRNAs were associated with cytokine-mediated signaling and regulation of inflammatory response (Fig. 2*D* and supplemental Table S3).

### miR-155 is synergistically induced by IFN-γ and TNF-α in HDLECs

**Figure 3.**
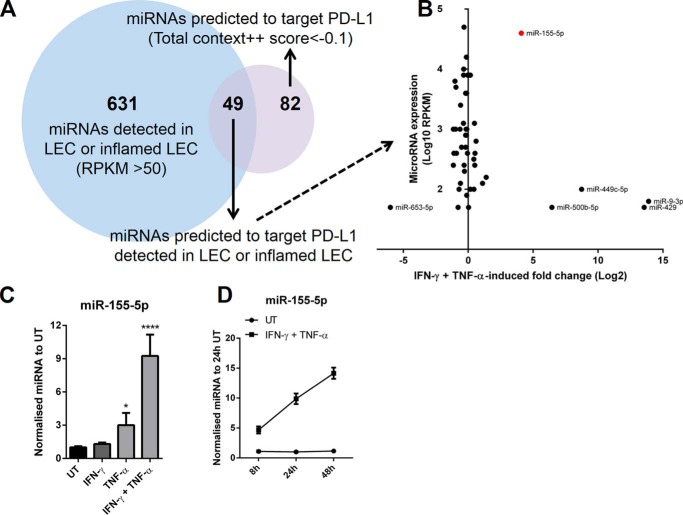
**miR-155 is synergistically induced by IFN-γ and TNF-α.** *A,* representing the overlap between the total number of detected miRNAs in HDLECs from small RNA sequencing and number of miRNAs predicted to target PD-L1 (TargetScan). *B,* comparison of the 49 miRNAs detected in LECs and predicted to target PD-L1 between average expression (log_10_ RPKM) and change in fold-expression after 24 h IFN-γ and TNF-α stimulation (log_2_). *C,* levels of miR-155 were measured by qRT-PCR after stimulation (24 h) with IFN-γ, TNF-α, or both, normalized to untreated. Statistical test used was one-way analysis of variance using Tukey's multiple comparisons test, *n* = at least 3 independent samples. *D,* time course of miR-155 expression following IFN-γ and TNF-α stimulation (8, 24, and 48 h), normalized to untreated (24 h), *n* = 3 independent samples. *, *p* < 0.05 and ****, *p* < 0.0001.

We compared detected miRNAs from small RNA sequencing with miRNAs predicted to target the 3′-UTR of PD-L1 using TargetScan software (26) (Fig. 3*A*). 49 detected miRNAs in basal or inflamed LECs also had predicted binding sites for PD-L1. Among these miRNAs, miR-155–5p (referred to as miR-155) was highly abundant and strongly induced by IFN-γ and TNF-α (Fig. 3*B*). As other highly induced miRNAs were lowly expressed we focused on miR-155 as a potential posttranscriptional regulator of PD-L1 expression in inflamed LECs. We dissected how miR-155 responded to IFN-γ and TNF-α and found that TNF-α was the primary inducer (Fig. 3*C*). Although IFN-γ alone did not affect miR-155 levels in HDLECs, it significantly enhanced the effect of TNF-α on miR-155 expression. Stimulating cells with increasing concentrations of IFN-γ in conjunction with TNF-α further increased miR-155 expression (supplemental Fig. S2). Up-regulation of miR-155 by IFN-γ and TNF-α was observed at 8 h and continued to rise, remaining at high levels after 48 h (Fig. 3*D*).

### miR-155 regulates PD-L1 expression after IFN-γ and TNF-α stimulation

**Figure 4.**
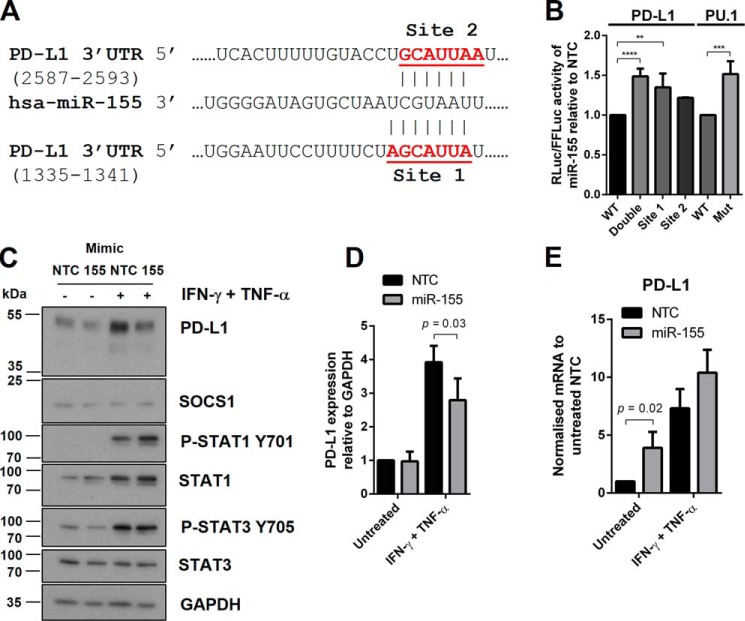
**miR-155 overexpression suppresses PD-L1 expression.** *A,* miR-155 has two binding sites on PD-L1 3′-UTR as predicted by TargetScan. *B,* relative *Renilla* luciferase (*RLuc*) to Firefly luciferase (*FFLuc*) activity for PD-L1 wild-type (*WT*) 3′-UTR, PD-L1 double mutant 3′-UTR, PD-L1 mutant 3′-UTR at 1335–1341 (Site 1) and 2587–2593 (Site 2), performed in HeLa cells transfected with miR-155 mimics (48 h). The WT and mutated 3′-UTR of PU.1, a known miR-155 target, was used as control. Statistical test used was one-way analysis of variance using Tukey's multiple comparisons test, *n* = 3–4 independent experiments, normalized to non-targeting control (*NTC*). *C,* protein expression following IFN-γ and TNF-α stimulation (24 h) in HDLECs transfected with miR-155 mimics (48 h). *D,* Western blot quantification of PD-L1 with miR-155 mimics, *n* = 3 independent experiments, normalized to untreated (*NTC*). *E,* PD-L1 mRNA expression measured by qRT-PCR following IFN-γ and TNF-α stimulation (24 h) in HDLECs transfected with miR-155 mimics (48 h), normalized to untreated (*NTC*). Statistical test (*D* and *E*) was unpaired Student's *t* test. *, *p* < 0.05; **, *p* < 0.01; ***, *p* < 0.001; ****, *p* < 0.0001.

We found two potential miR-155-binding sites on the 3′-UTR of PD-L1 (Fig. 4*A*) that are conserved in human and mice. To determine direct regulation of PD-L1 by miR-155, luciferase reporter assays were performed with mutagenesis of miR-155-binding sites. Wild-type, single, or double mutated miR-155-binding sites were co-transfected with miR-155 mimics in HeLa cells (Fig. 4*B*). Mutating the first miR-155-binding site (Site 1) in PD-L1 3′-UTR led to a significant increase in luciferase reporter activity compared with wild-type 3′-UTR, and the effect was more profound after mutating both miR-155-binding sites (Fig. 4*B*). Constructs containing the PU.1 3′-UTR, a previously validated miR-155 target (27), were used as controls for these assays. Next, we overexpressed miR-155 by transfecting miR-155 mimics into HDLECs (supplemental Fig. S3*A*). Overexpression of miR-155 resulted in significant down-regulation of 24 h IFN-γ- and TNF-α-induced PD-L1 expression (Fig. 4, *C* and *D*). STAT1 expression and phosphorylation of STAT1 were significantly increased (Figs. 4*C* and supplemental Fig. S3*B*). PD-L1 mRNA levels were consistently increased from overexpression of miR-155 (Fig. 4*E*).

**Figure 5.**
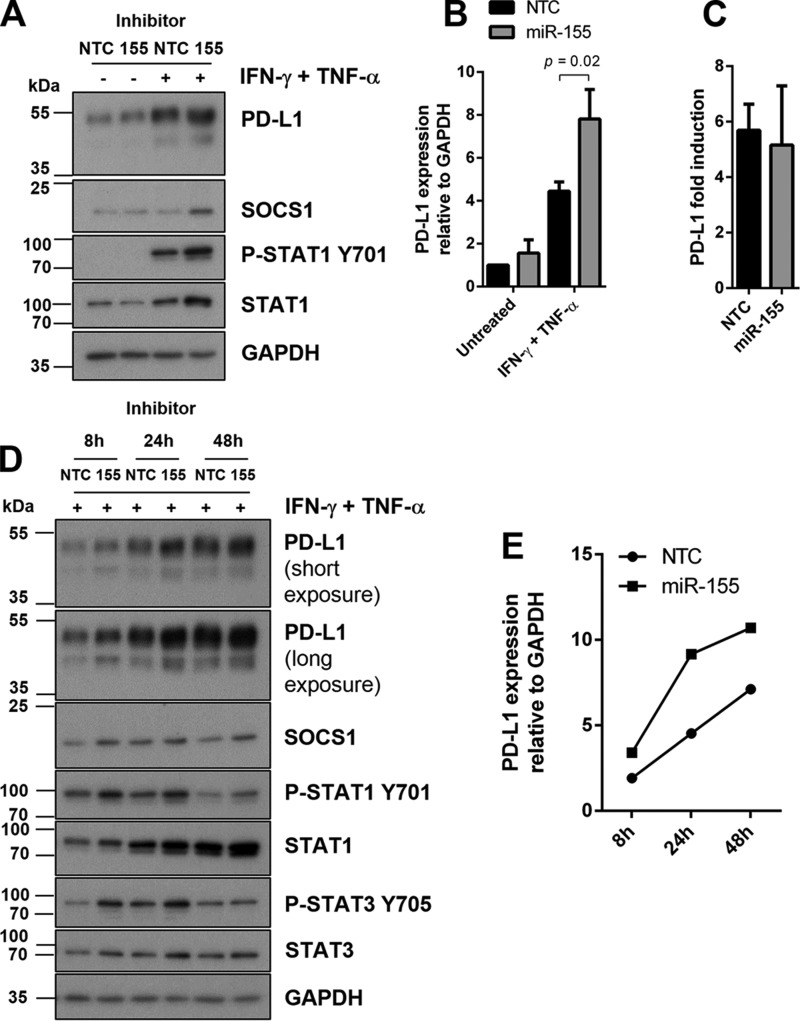
**Inhibition of miR-155 results in increased PD-L1 expression after IFN-γ and TNF-α stimulation.** *A,* protein expression following IFN-γ and TNF-α stimulation (24 h) in HDLECs transfected with miR-155 inhibitors (48 h). *B,* Western blot quantification of PD-L1 with miR-155 inhibitors, *n* = 3 independent experiments, normalized to untreated (*NTC*). Statistical test was unpaired Student's *t* test. *C,* PD-L1 mRNA fold-induction following IFN-γ and TNF-α stimulation (24 h) in HDLECs transfected with miR-155 inhibitors (48 h). *D,* protein expression following IFN-γ and TNF-α stimulation (8, 24, and 48 h) in HDLECs transfected with miR-155 inhibitors (48 h). *E,* Western blot quantification of time course from *D* showing expression of PD-L1 after transfection of miR-155 inhibitors.

**Figure 6.**
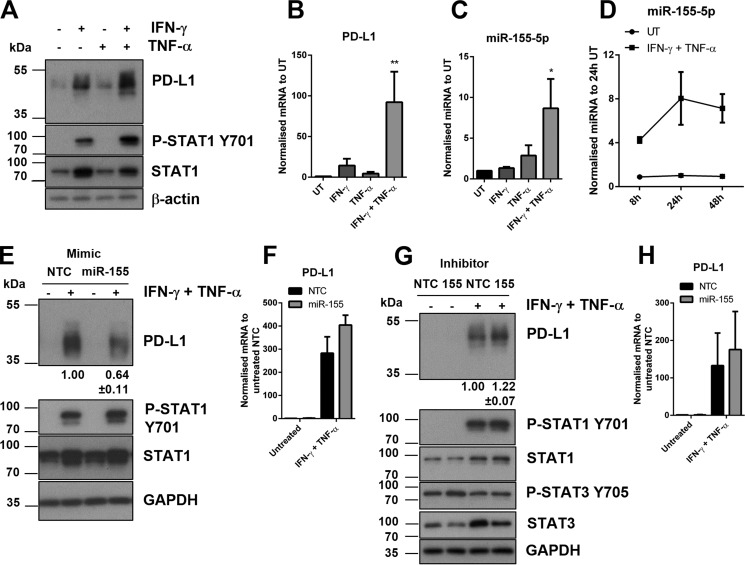
**miR-155 regulates PD-L1 in human dermal fibroblasts.** *A,* Western blot analysis following 24 h stimulation of HDFs with IFN-γ and TNF-α. *B,* PD-L1 mRNA levels measured by qRT-PCR after stimulation (24 h), normalized to untreated. One-way analysis of variance was calculated with Tukey's multiple comparisons test, **, *p* < 0.01. *C,* miR-155 expression measured by qRT-PCR following stimulation (24 h), normalized to untreated. Statistical test used was one-way analysis of variance using Tukey's multiple comparisons test. *D,* time course of miR-155 expression following IFN-γ and TNF-α stimulation (8, 24, and 48 h), normalized to untreated (24 h), *n* = 3 independent samples. *E,* protein expression following IFN-γ and TNF-α stimulation (24 h) in HDFs transfected with miR-155 mimics (48 h). *F,* PD-L1 mRNA expression following IFN-γ and TNF-α stimulation (24 h) in HDLECs transfected with miR-155 mimics (48 h). *G,* protein expression following IFN-γ and TNF-α stimulation (24 h) in HDFs transfected with miR-155 inhibitors. *H*, PD-L1 mRNA expression following IFN-γ and TNF-α stimulation (24 h) in HDLECs transfected with miR-155 inhibitors (48 h). Western blot quantification of PD-L1 normalized to IFN-γ and TNF-α treated NTC with standard deviation (*E* and *G*).

Next, we tested whether endogenous miR-155 could suppress PD-L1 expression. Inhibition of miR-155 resulted in significant up-regulation of IFN-γ and TNF-α-induced PD-L1 expression (Fig. 5, *A* and *B*). Suppressor of cytokine signaling 1 (SOCS1), a published target of miR-155 (28, 29) was noticeably increased after inhibition of miR-155 (Fig. 5*A*), although there was no change upon overexpression of miR-155 (Fig. 4*C*). Expression of STAT1 was increased in IFN-γ- and TNF-α-stimulated cells and decreased in untreated cells but resulted overall in no change in phosphorylation of STAT1 relative to total STAT1 (Fig. 5*A* and supplemental Fig. S4*A*). There was no change in the fold-induction of PD-L1 mRNA after IFN-γ and TNF-α stimulation, compared with control (Fig. 5*C*). To determine the effect of miR-155 on PD-L1 expression over time, we introduced an earlier (8 h) and later (48 h) time point for IFN-γ and TNF-α stimulation (Fig. 5, *D* and *E*, and supplemental Fig. S4*B*). Expression of PD-L1 at all three time points was increased following inhibition of miR-155 compared with control. This demonstrated that lack of miR-155 could affect the onset and maximum levels of PD-L1 expression upon IFN-γ and TNF-α treatments. Inhibition of miR-155 also resulted in the increased expression of SOCS1 and STAT1 that were consistently reproducible at the 24-h time point, and also of STAT3. To determine whether miR-155 targeting of PD-L1 could occur in a different cell type, we tested our model in primary HDFs. PD-L1 was undetectable at basal levels in HDFs (Fig. 6, *A* and *B*). However, we found PD-L1 to be inducible, along with IL-1β and miR-155 in similar synergistic activation following IFN-γ and TNF-α stimulation (Fig. 6, *A–C*, and supplemental Fig. S5). In treated HDFs, miR-155 was up-regulated within 8 h of stimulation, reaching its peak levels by 24 h (Fig. 6*D*). Overexpression of miR-155 resulted in PD-L1 down-regulation in activated HDFs (Fig. 6*E*) without a statistically significant effect on PD-L1 mRNA (Fig. 6*F*). Conversely, inhibition of miR-155 led to an increase of PD-L1 expression (Fig. 6*G*). miR-155 inhibition did not result in a statistically significant effect on PD-L1 mRNA under these conditions (Fig. 6*H*). These results indicate that miR-155-mediated suppression of PD-L1 is not specific to HDLECs and could be observed in other dermal primary cells responding to inflammatory stimuli.

## Discussion

**Figure 7.**
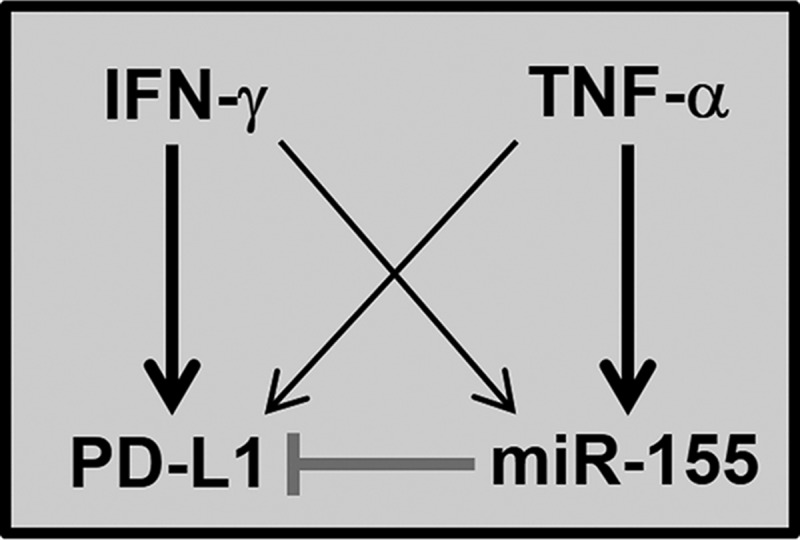
**Proposed schematic of miR-155-mediated regulation of PD-L1 in primary dermal cells responding to IFN-γ and TNF-α**. IFN-γ and TNF-α cooperate to induce PD-L1 expression in HDLECs and HDFs in a synergistic manner. In parallel, the two cytokines lead to induction of miR-155, which suppresses PD-L1 expression through canonical miRNA/mRNA targeting. *Thick arrows* indicate the predominant cytokine driving PD-L1 or miR-155 expression.

We reveal that in addition to promoting PD-L1 expression TNF-α and IFN-γ concurrently lead to induction of PD-L1-targeting miRNAs during a physiological immune response (Fig. 7). We identify miR-155 as a critical posttranscriptional PD-L1 regulator that limits maximal levels of PD-L1 expression in dermal cells responding to inflammation, but also the kinetics of the PD-L1 induction. Despite its central role in immune responses, there is only one study investigating posttranscriptional regulation of PD-L1 in non-cancer cells. miR-513 is down-regulated in a STAT1-dependent manner in IFN-γ-treated human biliary epithelial cells (30). This is reminiscent of suppression of PD-L1-targeting miRNAs in cancer cells to allow PD-L1 expression (19–21). In contrast, our results reveal that inflammation-induced miRNAs are crucial components of regulatory loops, which control PD-L1 expression to avoid excessive or prolonged PD-L1-mediated immunosuppression.

It is of note that miR-155, a multifunctional modulator of inflammation, innate and adaptive immunity, suppresses PD-L1 expression. miR-155-mediated silencing of PD-L1 in HDLECs and HDFs were consistently observed at the protein but not at the mRNA level. This might be explained by concurrent miR-155-mediated regulation of other genes affecting levels of PD-L1 mRNA (*e.g.* components of the JAK/STAT pathway) or by miR-155 primarily blocking PD-L1 translation rather than causing PD-L1 mRNA degradation. miR-155 is involved in development and function of T, B, and myeloid cells (27, 31, 32). Transcribed from a highly conserved non-coding B cell integration cluster on chromosome 21, miR-155 is expressed in myeloid and lymphoid cells (32). In several types of cancer, miR-155 is often abnormally expressed and associated with poor prognosis. As such, miR-155 is regarded as an oncogenic miRNA in B cell lymphoma and several solid tumors, including breast, lung, and colon (33–35). miR-155 is characterized as a primary component of the inflammatory response whereby a broad range of inflammatory mediators including bacterial lipopolysaccharide (LPS), poly(I:C) and TNF-α activate miR-155 in human and mice (16, 17, 36, 37). Interestingly, stimulation from IFN-β and IFN-γ also activate miR-155 and this was found to be dependent on TNF receptor type 1 (TNFR1) and JNK signaling (16). Furthermore, the JAK-STAT pathway is involved in up-regulation of IFN-γ and TNF-α-induced miR-155 expression in human retinal pigment epithelial cells (38). Our findings indicate that TNF-α, a cytokine that is up to now associated with posttranslational PD-L1 stabilization (11), drives induction of miR-155, which suppresses PD-L1 expression.

The interaction between miR-155 and PD-L1 reveals the existence of a complex regulatory network. SOCS1, a negative feedback regulator of IFN-γ/STAT signaling, which inhibits JAK tyrosine kinase activity, has been shown to be a direct target of miR-155 in human and mice (28, 29, 39–41). In some cases, miR-155 expression is inversely correlated with SOCS1, as shown in breast cancer (39). Overexpression of miR-155 has been shown to decrease SOCS1 and increase phosphorylation levels of JAK2 and phospho-STAT3 (39–41). Interestingly, miR-155 suppresses expression of SOCS1 leading to enhanced STAT1 phosphorylation in macrophages (29), miR-155-deficient CD8^+^ T cells display enhanced levels of STAT1 phosphorylation (42). We observed that inhibition of miR-155 increased SOCS1 expression, although overexpression of miR-155 did not affect SOCS1 levels in HDLECs. Moreover, we found no increase in STAT3 after miR-155 overexpression but rather an increase in phospho-STAT3 at 8 h after inhibiting miR-155. Additionally, we found that overexpression and inhibition of miR-155 both increased the levels of STAT1 indicating the existence of dose-dependent effects, in agreement with previous reports (42, 43). Based on the above, we propose that miR-155 affects the JAK/STAT pathway through multiple mechanisms, likely in a cell type-specific manner. Nevertheless, the observed effects of miR-155 mimics and inhibitors on PD-L1 protein expression in combination with the direct binding of the miRNA to the PD-L1 3′-UTR, demonstrate that direct targeting of PD-L1 by miR-155 is a crucial component of the cytokine receptor (IFNGR or TNFR)/JAK/STAT/SOCS1/miR-155/PD-L1 network.

Overall, our study provides a novel perspective on the posttranscriptional regulation of PD-L1 during inflammation. We reveal a number of potentially PD-L1-targeting miRNAs as responsive to inflammatory challenge. These include miR-155, which plays a primary role in inflammation and can be induced by a broad range of inflammatory mediators. Concomitantly, we show that PD-L1 is induced upon inflammation and contributes toward immune suppression in HDLECs expanding previous findings in macrovascular endothelial cells (5, 22). Furthermore, we show that in dermal vascular and stromal cells, miR-155 acts to suppress PD-L1 induction to fine-tune the immune response. As miR-155 is expressed by a variety of immune cells and frequently overexpressed in cancer, we propose that our findings have broad implications in our understanding of PD-L1 expression in a variety of physiological and disease contexts.

## Experimental procedures

### Cell culture and reagents

Primary HDLEC were purchased from Promocell and grown in endothelial cell growth media MV (Promocell) supplemented with vascular endothelial growth factor C (VEGF-C) (R&D Systems) (18). All experiments with HDLEC were performed at passage 5. Primary HDFs were grown in DMEM supplemented with 10% FCS, 1% l-glutamine, and 1% penicillin/streptomycin. Human recombinant IFN-γ was obtained from PeproTech and TNF-α was purchased from R&D Systems.

### RNA interference and miRNA inhibitors and mimics

Cells were seeded in 6-well plates 1 day before transfection with miRIDIAN hsa-miR-155–5p mimic (25 nm), hsa-miR-155–5p hairpin inhibitor (50–100 nm) (GE Dharmacon) based on the mature hsa-miR-155–5p sequence (5′-UUAAUGCUAAUCGUGAUAGGGGU-3′) or siRNAs targeting PD-L1 (50 nm, On-TargetPlus Smartpool, GE Dharmacon) using TransIT-siQuest transfection reagent (Mirus Bio). All experiments utilized respective negative controls (GE Dharmacon). 48 h posttransfection, cells were stimulated with IFN-γ and TNF-α for 24 h and harvested for experimental analysis.

### RNA isolation and qRT-PCR

Total RNA was isolated using the miRNeasy Kit (Qiagen). PD-L1 and IL-1β mRNA expression were quantified by qRT-PCR using SYBR Green Master Mix (Applied Biosystems). β-Actin was monitored as a housekeeping reference gene. The following primers were used at a final concentration of 300 nm: PD-L1 (F), 5′-CATCTTATTATGCCTTGGTGTAGCA-3′ and (R) 5′-GGATTACGTCTCCTCCAAATGTG-3′; IL-1β (F), 5′-AGGATGAC-TTGTTCTTTGAAGCTGA-3′ and (R), 5′-TGCCTGAAGCCCTTGCTG-3′; β-actin (F), 5′-CACCATTGGCAATGAGCGGTTC-3′ and (R), 5′-AGGTCTTTGCGGATGTCCACGT-3′. Commercially available primers (Applied Biosystems) were used to assess mature miRNA levels and the loading control U6 snRNA. Relative gene expression was calculated by the comparative *C_T_* method.

### Small RNA sequencing

RNA were isolated and enriched for small RNA using the PureLink miRNA isolation kit (Ambion). RNA integrity was assessed using the Agilent 2100 Bioanalyzer (Agilent Technologies). Sequencing libraries were generated using NEBNext Multiplex Small RNA Library Prep Set for Illumina (Set 1) (New England Biolabs) according to the manufacturer's instructions. Samples were sequenced using Illumina MiSeq (pair ended, 75 bp, MiSeq version 3). Sequencing reads were examined for quality and mapped against all annotated human mature and precursor miRNA sequences (miRBase version 21.0). Residual adapter sequences and indexes were removed with Cutadapt (version 1.8.3), in the paired-end mode, first trimming any low-quality ends with a cutoff of Q10 (−q 10), then removing flanking Ns (−trim-*n*) and any reads with >20% Ns (−max-*n* 0.2). Reads were quality trimmed with Sickle (version 1.330), with a cutoff of >Q20 (−q 20), and truncating at the position of the first N (−n). Reads were mapped with Bowtie (version 1.0.1) with a seed length of 15 (−l 15), a maximum total quality score at mismatched positions of 99999 (−e 99999), reporting all valid alignments per read or read pair (−a), and the best option to pick the best reported alignments. Reads were mapped separately for merged reads and a concatenated file of unmerged forward and reverse reads. Reads were counted using Subread featureCounts (version 1.5.0-p1), with a minimum fragment length of 5 (−d 5). Reads were counted against all features in the HsGRCh38 GFF file as well as against features from mirBASE release 21. Counts for the mapped merged reads were doubled and then added to the counts for the mapped unmerged reads. Duplicate features, *i.e.* those with identical numbers of mapped reads across all samples and identical lengths, were removed. RPKMs (reads per kilobase transcript per million mapped reads) were calculated and then log_2_-transformed and 75th percentile shifted. Reads mapping to protein coding or pseudogenes were presumed to correspond to degraded RNA and were excluded from descriptive analyses of data (Fig. 2*A*). For each feature across all of the samples the baseline was set to the median value (*i.e.* the median subtracted from all of the values for that feature). A two-tailed *t* test and FDR *p* value correction were used to assess statistical significance.

### Western blot analysis

Cells were lysed with ice-cold radioimmunoprecipitation assay (RIPA) buffer (5 mm EDTA, 150 mm NaCl, 10 nm Tris-HCl, pH 7.2, 0.1% SDS, 0.1% Triton X-100, and 1% sodium deoxycholate) containing protease mixture inhibitors P8340, P5726, and P0044 (Sigma). Protein concentration was determined by bicinchoninic acid assay (BCA) (Thermo Scientific) according to the manufacturer's protocol, using BSA as standards. Protein samples were denatured and resolved on SDS-PAGE gels using a Bio-Rad PowerPac HC and transferred onto PVDF membranes (Millipore). Membranes were probed overnight at 4 °C (1:1000) for the following primary antibodies to PD-L1 (E1L3N), SOCS1 (A156), STAT1 (9172), P-STAT1 Tyr-701 (D4A7), STAT3 (9132), and P-STAT3 Tyr-705 (D3A7) all from Cell Signaling, and for 1 h at room temperature for GAPDH (6C5) and β-actin (ab6276), both from Abcam. Membranes were further incubated with horseradish peroxidase (HRP)-conjugated secondary antibodies and visualized with ECL (GE Healthcare). Band intensity was quantified using ImageJ version 1.50e (NIH, Bethesda, MD).

### PNGase F treatment

Peptide:*N*-glycosidase F (PNGase F) was acquired from New England Biolabs (P0704). PNGase F was added to denatured protein lysates according to the manufacturer's protocol and subsequently analyzed by Western blot.

### Luciferase assays

PD-L1 3′-UTR were amplified from HeLa and subcloned into the psiCheck2 vector using XhoI and PmeI enzymes. Mutations were introduced at the PD-L1 3′-UTR at the miR-155-binding site (Site 1, 5′-AGCAUUA-3′ to 5′-UCUACAG-3′ and Site 2, 5′-GCAUUAA-3′ to 5′-UCUACAG-3′) using a Q5 site-directed mutagenesis kit (New England Biolabs) and confirmed by DNA sequencing. The PU.1 3′-UTR constructs were described previously (27). Luciferase assays were performed in HeLa cells transfected with hsa-miR-155–5p mimic (50 nm) and PD-L1 or PU.1 3′-UTR constructs for 48 h using JetPrime reagent. Samples were assayed with the Dual Luciferase reporter assay system kit (Promega) for Firefly and *Renilla* luciferase activities and measured on a PerkinElmer Life Sciences Wallac Victor2 1420 multilabel counter.

### Flow cytometry

Cells were incubated with anti-PD-L1 (5H1, kindly provided by Dr. Lieping Chen's laboratory, Yale University) and visualized with Brilliant Violet 421 (BioLegend) on a BD LSR Fortessa (BD Biosciences) using FACS DIVA software. Final analysis was done using FlowJo version 10 (Tree Star).

### Microscopy

Cells were cultured in 35-mm glass-bottom dishes with a 14-mm microwell (MatTek). After 48 h, cells were stimulated with IFN-γ or in combination with TNF-α for 24 h. Cells were fixed in 4% paraformaldehyde and permeabilized with 0.5% Triton X-100. Samples were incubated overnight at 4 °C with anti-PD-L1 (5H1) followed by goat anti-mouse secondary Alexa Fluor 488 (Thermo Scientific) and DAPI was used to stain the nucleus. Images were acquired with Zeiss Zen software using a Zeiss LSM 880 on a ×40 oil immersion objective lens.

### Statistical analysis

Experimental results are presented as mean ± S.D. The specific statistical tests are mentioned in the figure legends. Statistical analysis and graphs were made using GraphPad Prism 6 (GraphPad Software).

## Author contributions

D. Y. performed and designed experiments and wrote the manuscript. K. M. S. performed experiments. M. C. C. contributed to experimental design and supervised research. T. V. S. contributed to experimental design, supervised research, and co-wrote the manuscript. D. L. conceived the project, designed experiments, supervised research, and wrote and edited the manuscript.

## Supplementary Material

Supplemental Data

## Footnotes

4 The abbreviations used are:

LEC: lymphatic endothelial cell
HDLEC: human dermal LECs
HDF: human dermal fibroblast
PD-L1: programmed death ligand-1
SOCS-1: suppressor of cytokine signaling 1
qRT: quantitative RT
PNGase F: peptide:*N*-glycosidase F
miRNA: microRNA
RPKM: reads per kilobase transcript per million mapped reads.
