# Supplementary material for: MicroRNA-155 induction via TNF-α and IFN-γ suppresses expression of programmed death ligand-1 (PD-L1) in human primary cells
Source: J Biol Chem. 2017 Oct 24;292(50):20683–93. doi: 10.1074/jbc.M117.809053 (PMC5733604; doi:10.1074/jbc.M117.809053)
Supplement: Supplemental Data [file supp_292_50_20683__index.html]

MicroRNA-155 induction via TNF-α and IFN-γ suppresses expression of programmed death ligand-1 (PD-L1) in human primary cells — miR-155 targets PD-L1 in human primary cells — Supplemental Data 

# MicroRNA-155 induction via TNF-α and IFN-γ suppresses expression of programmed death ligand-1 (PD-L1) in human primary cells

## Supplemental Data

- Supplemental Figures and Table S2 (.pdf, 880 KB) - Supplemental Figures and Table S2
- Supplemental Table S1 (.xlsx, 740 KB) - Supplemental Table S1
- Supplemental Table S3 (.xlsx, 284 KB) - Supplemental Table S3
