# Supplementary material for: MicroRNA-155 induction via TNF-α and IFN-γ suppresses expression of programmed death ligand-1 (PD-L1) in human primary cells
Source: J Biol Chem. 2017 Oct 24;292(50):20683–93. doi: 10.1074/jbc.M117.809053 (PMC5733604; doi:10.1074/jbc.M117.809053)
Supplement: Supplemental Data [file 10.1074_M117.809053_jbc.M117.809053-1.pdf]

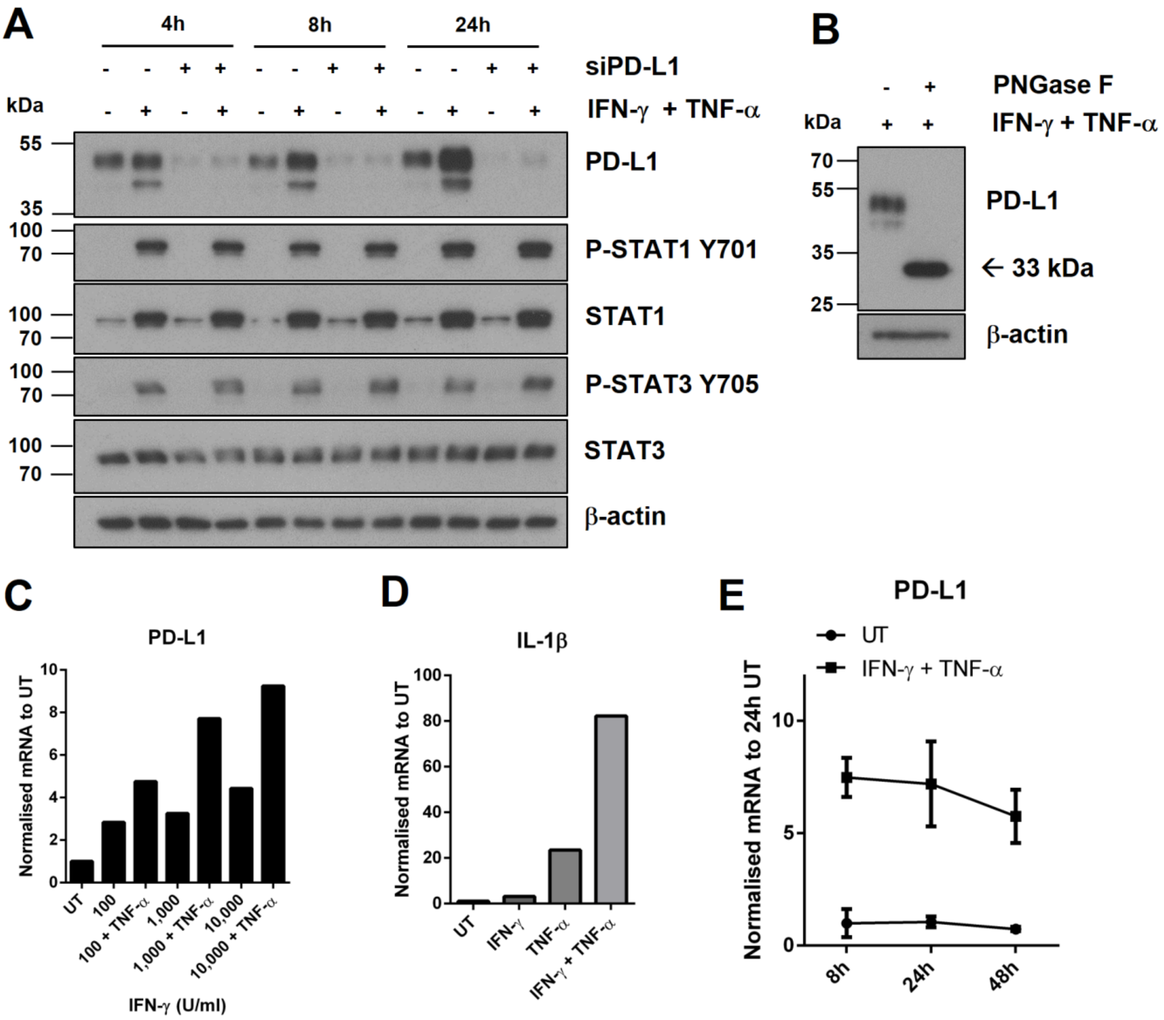

Figure S1. Specific expression of PD-L1 in HDLECs. (A) Western blot analysis following 48 h transfection of siRNA targeting PD-L1. Cells were stimulated with IFN-γ and TNF-α for 4, 8 and 24 h after siRNA transfection. (B) IFN-γ and TNF-α stimulated lysates were treated with PNGase F and analyzed by western blot. (C) qRT-PCR showing PD-L1 mRNA expression in cells treated with titrating amounts of IFN-γ with or without TNF-α for 24 h. (D) qRT-PCR measuring IL-1β levels following 24 h stimulation. (E) qRT-PCR showing time-course of PD-L1 mRNA expression in cells treated with IFN-γ and TNF-α for 8, 24 and 48 h and normalized to untreated (24 h).

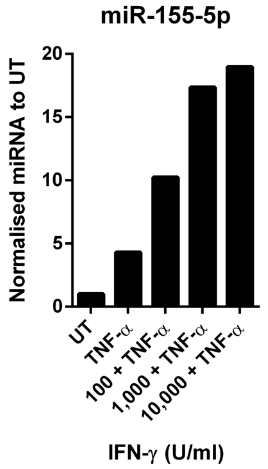

Figure S2. miR-155 expression increases with IFN-γ dosage. (A) miR-155 was measured by qRT-PCR in HDLECs stimulated (24 h) with the combination of TNF-α and increasing ten-fold doses of IFN-γ.

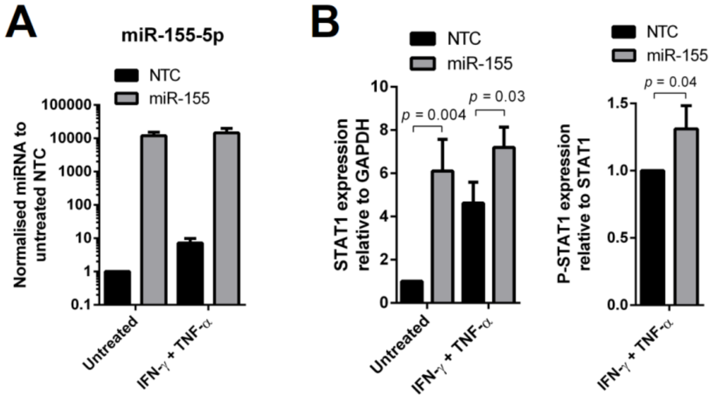

Figure S3. miR-155 overexpression affects STAT1. HDLECs were transfected with miR-155 mimics (48 h) and treated with IFN-γ and TNF-α (24 h) (see Fig. 4C-E for context). (A) miR-155 levels measured by qRT-PCR. (B) Western blot quantification of STAT1 and P-STAT1/STAT1. Statistical test was unpaired Student's t-test.

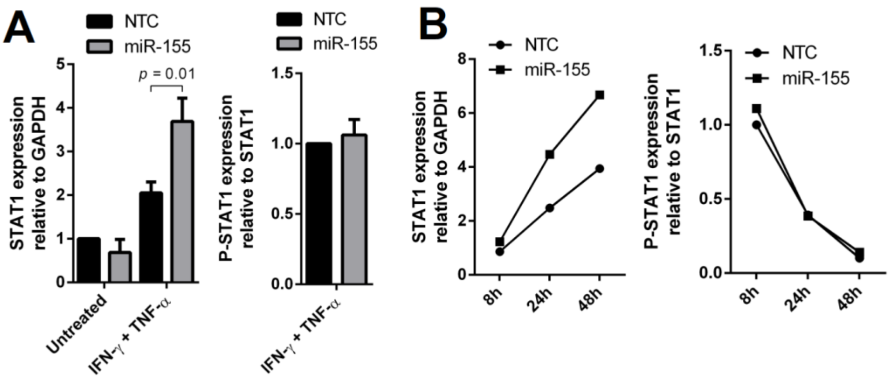

Figure S4. miR-155 inhibition affects induced STAT1 expression but not phosphorylation. HDLECs were transfected with miR-155 inhibitors (48 h) and treated with IFN-γ and TNF-α (24 h). (A) Western blot quantification of STAT1 and P-STAT1/STAT1 (see Fig. 5A for context). Statistical test was unpaired Student's t-test. (B) Western blot

quantification showing time-course expression of STAT1 and P-STAT1/STAT1 following IFN-γ and TNF-α stimulation for 8, 24 and 48 h (see Fig 5D).

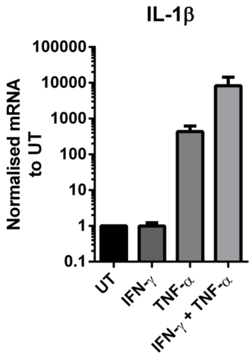

Figure S5. IFN-γ and TNF-α synergistically upregulate IL-1β in fibroblasts. (A) HDFs were stimulated with IFN-γ and TNF-α for 24 h and IL-1β mRNA was measured by qRT-PCR.

Table S1. Sequencing data.

miRNA	Sequencing fold change	qRT-PCR fold change
miR-582-5p	0.36	0.38
miR-582-3p	0.35	0.36
miR-671-5p	0.45	0.82
miR-216a-5p	0.49	0.72
miR-93-5p	0.52	0.67
miR-217	0.60	0.66
miR-125b-5p	0.60	0.49
miR-146a-5p	3.33	2.56
miR-218-5p	3.43	2.99
miR-4485-3p	6.57	1.51
miR-155-5p	17.03	17.16

Table S2. Validation of IFN-γ and TNF-α-regulated targets. Table showing data from small RNA sequencing and qRT-PCR (as shown in Fig. 2B and 2C). Values represent average fold change of miRNA expression following IFN-γ and TNF-α stimulation (24 h) in HDLECs.

Table S3. miRNA enrichment analysis and annotation tool (miEAA) data.
